# Arboviral disease record data - Dengue and Chikungunya, Brazil, 2013–2020

**DOI:** 10.1038/s41597-022-01312-7

**Published:** 2022-05-10

**Authors:** Sebastião Rogério da Silva Neto, Thomás Tabosa de Oliveira, Igor Vitor Teixiera, Leonides Medeiros Neto, Vanderson Souza Sampaio, Theo Lynn, Patricia Takako Endo

**Affiliations:** 1 Universidade de Pernambuco, Programa de Pós-Graduação em Engenharia da Computação, Recife, 50720-001 Brazil; 2 Fundação de Medicina Tropical Dr. Heitor Vieira Dourado, Manaus, 69040-000 Brazil; 3 Instituto Todos pela Saúde, São Paulo, 01310-942 Brazil; 4 Irish Institute of Digital Business, Dublin City University, Dublin, 9 Ireland

**Keywords:** Public health, Diseases

## Abstract

One of the main categories of Neglected Tropical Diseases (NTDs) are arboviruses, of which Dengue and Chikungunya are the most common. Arboviruses mainly affect tropical countries. Brazil has the largest absolute number of cases in Latin America. This work presents a unified data set with clinical, sociodemographic, and laboratorial data on confirmed patients of Dengue and Chikungunya, as well as patients ruled out of infection from these diseases. The data is based on case notification data submitted to the Brazilian Information System for Notifiable Diseases, from Portuguese *Sistema de Informação de Agravo de Notificação* (SINAN), from 2013 to 2020. The original data set comprised 13,421,230 records and 118 attributes. Following a pre-processing process, a final data set of 7,632,542 records and 56 attributes was generated. The data presented in this work will assist researchers in investigating antecedents of arbovirus emergence and transmission more generally, and Dengue and Chikungunya in particular. Furthermore, it can be used to train and test machine learning models for differential diagnosis and multi-class classification.

## Abstract (summary)

**Table:** 

Measurement(s)	clinical data
Technology Type(s)	interview

## Background & Summary

Arboviral diseases are a global health concern due to their rapid geographic spread. These diseases are transmitted through arthropod insects such as *Aedes Aegypti* and *Aedes Albopictus*. These types of virus, known as arboviruses, are more commonly found in tropical countries whose climates favour viral amplification and transmission^1^. Among these diseases, Dengue, Chikungunya, Yellow Fever, and, more recently, Zika, have higher prominence due to their relatively higher case numbers. Over the past thirty years, the spread and impact of these diseases on public health have increased dramatically^2^. Furthermore, there is evidence that COVID-19 intervention measures, such as lockdowns, have contributed to an increase in arbovirus cases^3^.

The spread of Dengue in recent decades is dramatic. In 2019, WHO Region of the Americas recorded the highest number of Dengue cases in history^4^. Brazil has the highest number of absolute cases of Dengue and Chikungunya worldwide^5,6^. These two diseases are the most common arboviral diseases in the country; both reached historical peaks in recent years. For example, reported cases and deaths due to Dengue reached a peak of 2,248,570 cases and 840 deaths in 2019^5^. In 2016, Brazil there were 558,542 reported cases of Chikungunya, the highest number reported to date^6^.

The correct diagnosis of arboviruses is a significant challenge. According to Pan American Health Organization (PAHO)^5,6^, only about half of reported cases are confirmed, with the remainder being treated as suspected cases. This is due to the concurrency of circulation of these diseases and the high similarity in the symptoms of Dengue and Chikungunya which makes clinical diagnosis difficult. In the absence of point-of-care virus-specific testing, even experienced and well-trained physicians may misdiagnose an arbovirus infection due to the similarity in symptoms^7^. Rapid tests, especially for Dengue, are effective in confirming the disease but only up to the fifth day post-infection. After this period, such tests have a high rate of error thus requiring the use of laboratory tests. Unfortunately, laboratory testing requires technical equipment that is not widely available throughout Brazil. In addition, laboratory testing is also subject to misdiagnosis due to co-infection and cross-reaction with the various arboviruses found in the country^8^. Such misdiagnosis can result in a wide range of negative outcomes including inadequate or inappropriate treatment. Indeed, despite arboviruses being notifiable diseases in Brazil and the public sector being the primary health service provider for over 70% of the population, relatively few confirmatory tests are carried out^7^. According to the Brazilian Ministry of Health^9^, “only approximately 23% were tested in reference laboratories”.

Given that Brazil is hyper-endemic for arboviruses, the amount of patient data collected is very large. For example, almost 1.5 million cases of Dengue were reported to Brazilian Information System for Notifiable Diseases, from Portuguese Sistema de Informacao de Agravo de Notificacao (SINAN) in 2020. As such, this represents a significant source of information for both epidemiological analysis as well as training and optimising machine learning models for health purposes. The objective of this work is to make available a Brazilian national data set with clinical, laboratory, and socio-demographic data on both confirmed, discarded, and inconclusive cases of Dengue and Chikungunya so that this data can be used for future research, such as the development of machine learning model that helps to correctly classify these patients. A high-level epidemiological analysis of the data set is also presented.

## Methods

The data was collected from the Brazilian Information System for Notifiable Diseases, *Sistema de Informação de Agravo de Notificação* (SINAN) http://portalsinan.saude.gov.br/. The data set is from a public data repository and according to current Brazilian laws, there is no need for ethics committee approval. SINAN collates case notification data of diseases present on the national list of compulsory notification of diseases, injuries and public health events https://bvsms.saude.gov.br/bvs/saudelegis/gm/2020/prt0264_19_02_2020.html. This includes Dengue and Chikungunya. The data contains notifications of Dengue and Chikungunya cases that occurred in Brazil, including all 26 states and the Federal District (*Brasília*), between 2013 and 2020. Dengue-related data contains clinical data (pre-existing symptoms and comorbidities), laboratory tests performed, and socio-demographic data for each case. With the exception of one hundred records, Chikungunya-related data contains only socio-demographic data. No explanation on why only one hundred Chikunya records contain clinical and laboratory test data was provided with the data. It is possible that these cases were treated as suspected cases of Dengue and only later confirmed as cases of Chikungunya however this has not been confirmed. These cases are included in the data set summary in Table 6. For both data sets, no individually identifiable health information is made available in the data set.

**Fig. 1:**
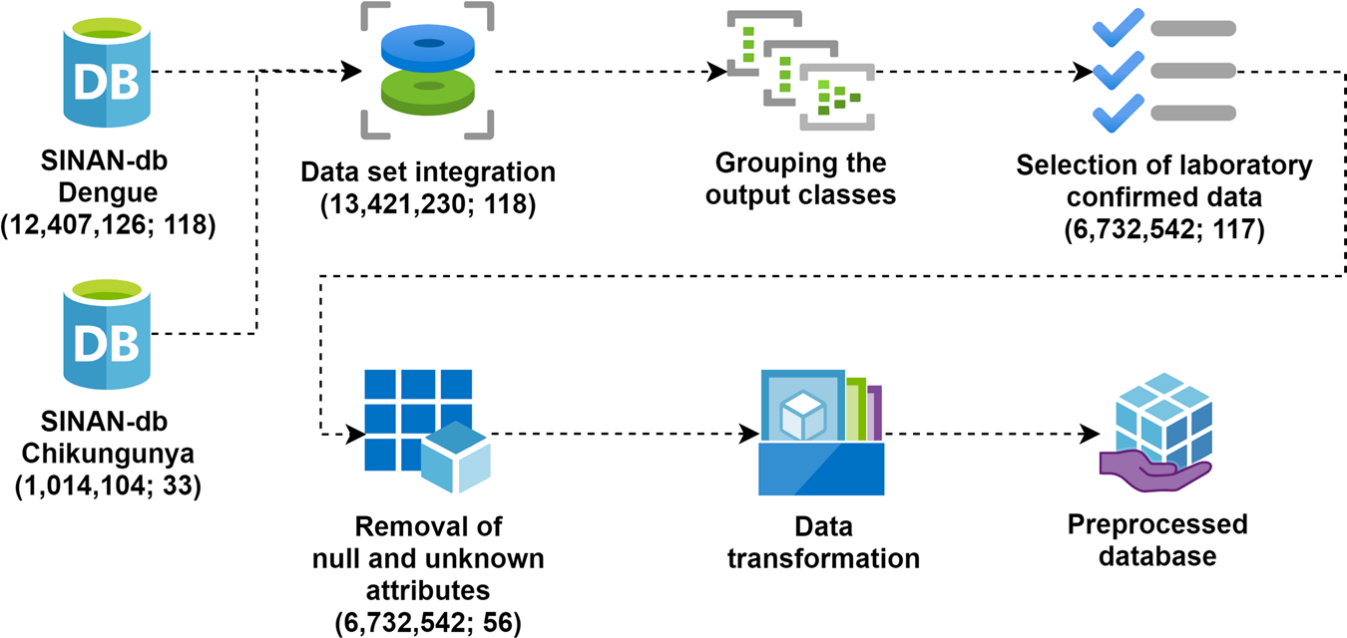
Pre-processing steps performed to build the final data set.

Figure 1 presents the preprocessing steps used for cleaning the data set. First, the SINAN data from all states were aggregated resulting in 13,421,230 notifications and 118 attributes. The records were grouped into three distinct groups by the CLASSI_FIN attribute:

- Dengue: Patients with confirmed Dengue;
- Chikungunya: Patients with confirmed Chikungunya; and
- Discarded/Inconclusive: Patients who tested negative or inconclusive for Dengue or Chikungunya following laboratory tests.

Only notifications that were (a) confirmed or (b) discarded/inconclusive following clinical diagnostic were selected. For confirmation criteria, we used the Brazilian MS definitions that can be found here: https://bvsms.saude.gov.br/bvs/publicacoes/diretriz_nacionais_prevencao_controle_dengue.pdf. After this step, the attribute used for the filter (CRITERIO) was also removed, since it now contains only a single value. The attribute TP_NOT identifies the type of notification generated. As all notifications are of the “Individual” type, the TP_NOT attribute has the same value for all records. Attributes that had more than 60% null data or that were not in the original data dictionary were also removed. Attributes that still had null fields were filled with the default value, “not informed”, as per the data dictionary. The transformation from categorical to numerical data was also carried out. Table 1 shows all the attributes removed in the preprocessing process.

**Table 1:** Attributes removed after preprocessing.

Attributes removed					
ID_OCUPA_N	DT_ALRM	DT_VIRAL	GRAV_METRO	DT_OBITO	PETEQUIAS
DT_CHIK_S1	GRAV_PULSO	DT_PCR	GRAV_SANG	ALRM_HIPOT	HEMATURA
DT_CHIK_S2	GRAV_CONV	SOROTIPO	GRAV_AST	ALRM_PLAQ	SANGRAM
DT_PRNT	GRAV_ENCH	DT_INTERNA	GRAV_MIOC	ALRM_VOM	LACO_N
RES_CHIKS1	GRAV_INSUF	GENGIVO	GRAV_CONSC	ALRM_SANG	PLASMATICO
RES_CHIKS2	GRAV_TAQUI	MUNICIPIO	GRAV_ORGAO	ALRM_HEMAT	EVIDENCIA
RESUL_PRNT	GRAV_EXTRE	COUFINF	DT_GRAV	ALRM_ABDOM	PLAQ_MENOR
DT_SORO	GRAV_HIPOT	COPAISINF	MANI_HEMOR	ALRM_LETAR	COMPLICA
DT_NS1	GRAV_HEMAT	COMUNINF	EPISTAXE	ALRM_HEPAT	
DT_VIRAL	GRAV_MELEN	DOENCA_TRA	CLINC_CHIK	METRO	
TP_SISTEMA	CS_FLXRET	TP_NOT	CRITERIO	ALRM_LIQ	

At the end of the process, the data set consisted of 4,307,513 records for Dengue, 325,000 records for Chikungunya, and 2,100,029 records for the Discarded/Inconclusive category.

## Data Records

**Fig. 2:**
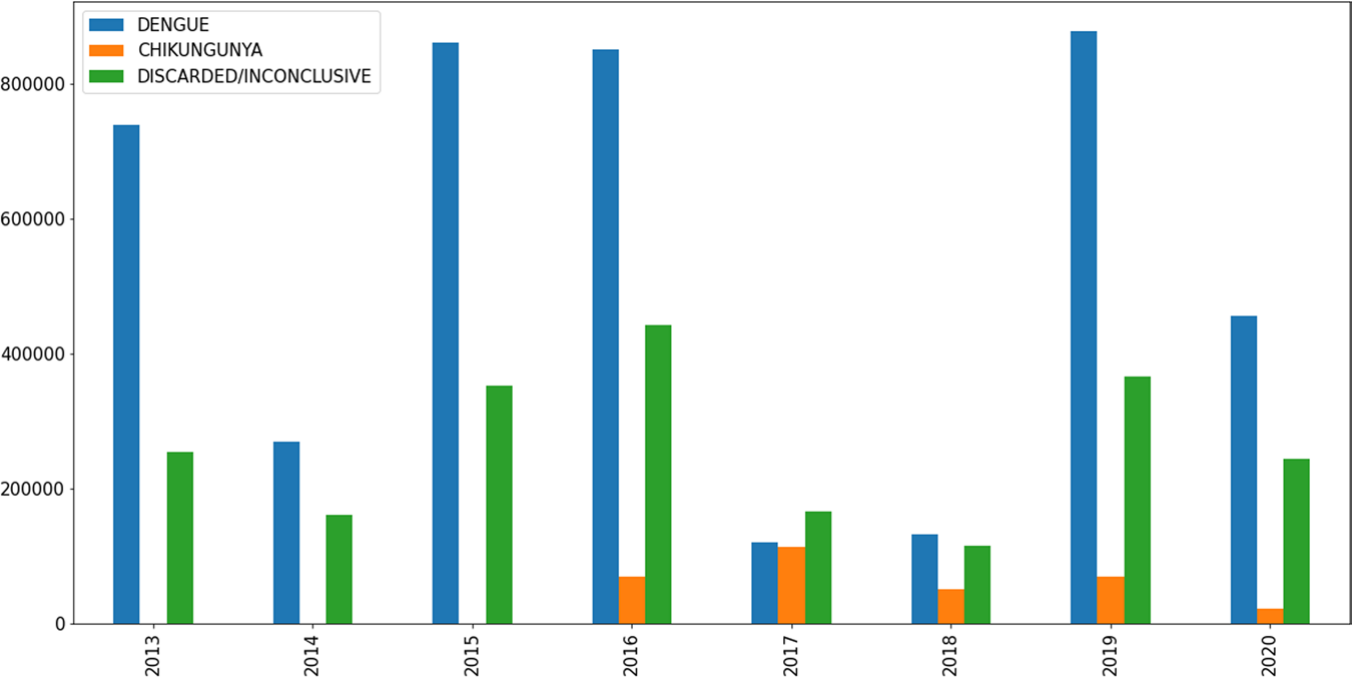
Number of records in the data set by category (Dengue, Chikungunya, Discarded/Inconclusive) in Brazil per year.

The processed data set, as well as the raw data, are available in Mendeley Data^10^ and can be found via the link https://data.mendeley.com/datasets/2d3kr8zynf/4. Figure 2 presents the number of records in the data set by category (Dengue, Chikungunya, Discarded/Inconclusive) in Brazil from 2013–2020. As can be clearly seen, Dengue infections in 2013, 2015, 2016, and 2019 were comparatively high^11,12^. In 2017, there was a drop in confirmed cases of both Dengue and Chikungunya in the country to similar levels for both diseases (120,753 cases of Dengue and 113,087 cases of Chikungunya).

**Fig. 3:**
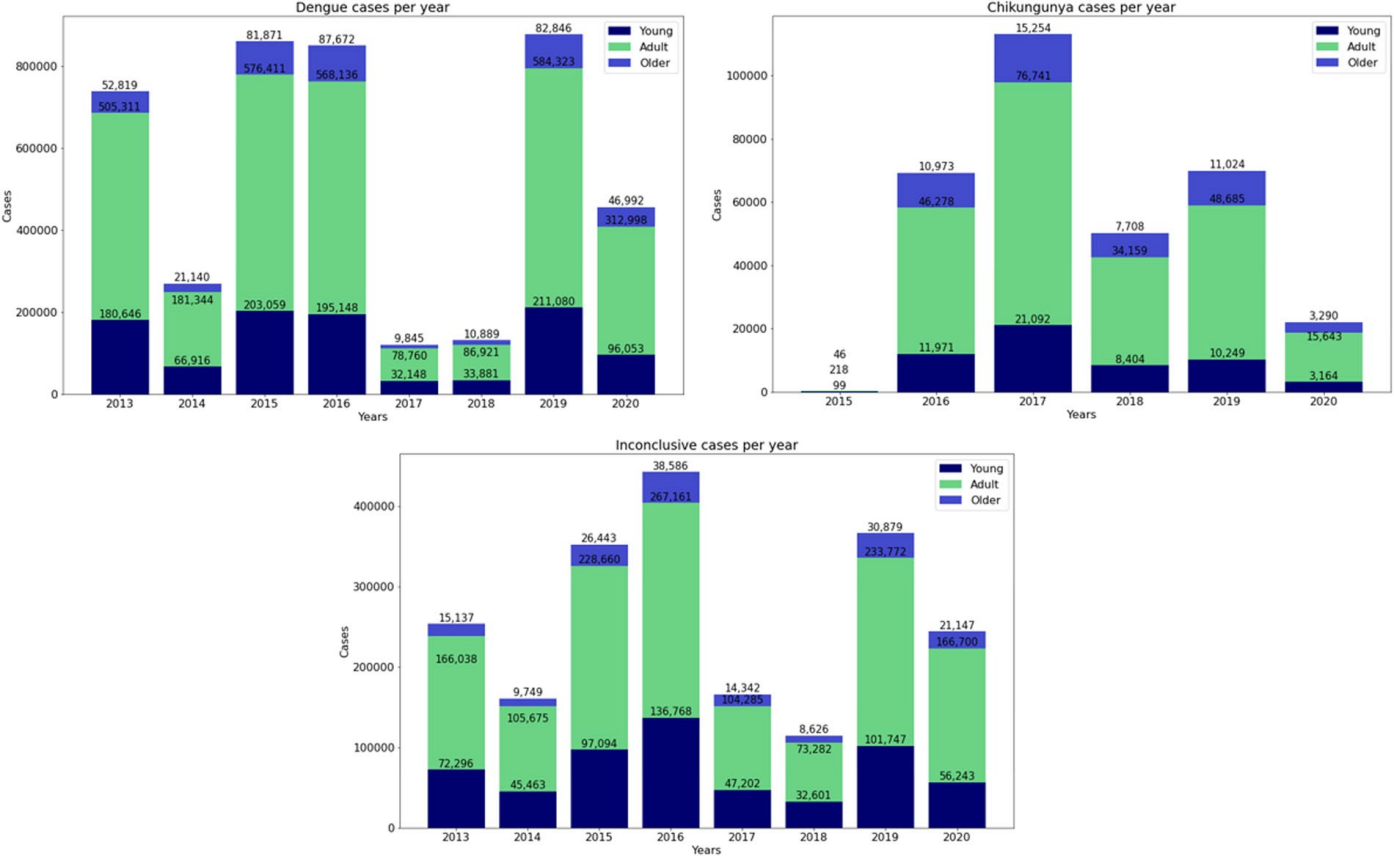
Age structure of individuals in cases of Dengue, Chikungunya and Inconclusive.

Figure 3 shows the age structure of the cases reported in this data set, divided into three categories: young people, adults and the elderly. The youth category includes individuals up to 18 years of age. The adult category is for individuals aged between 20 and 59 years. Finally, the elderly category are individuals aged 60 and over. In every year, the highest incidence of Dengue, Chikungunya or Inconclusive cases is in the adult category.

**Fig. 4:**
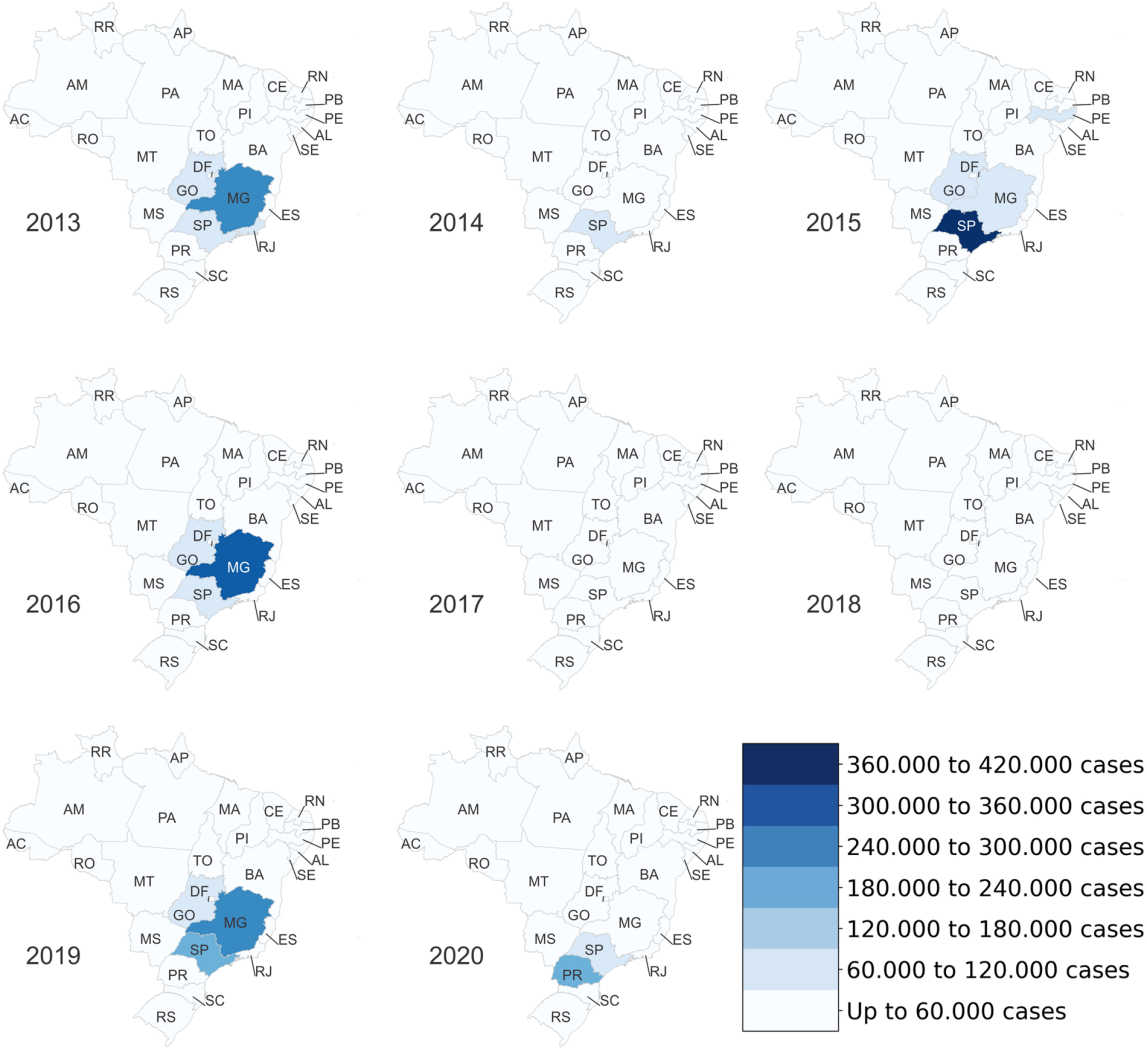
Occurrence of confirmed cases of Dengue by Brazilian state.

**Fig. 5:**
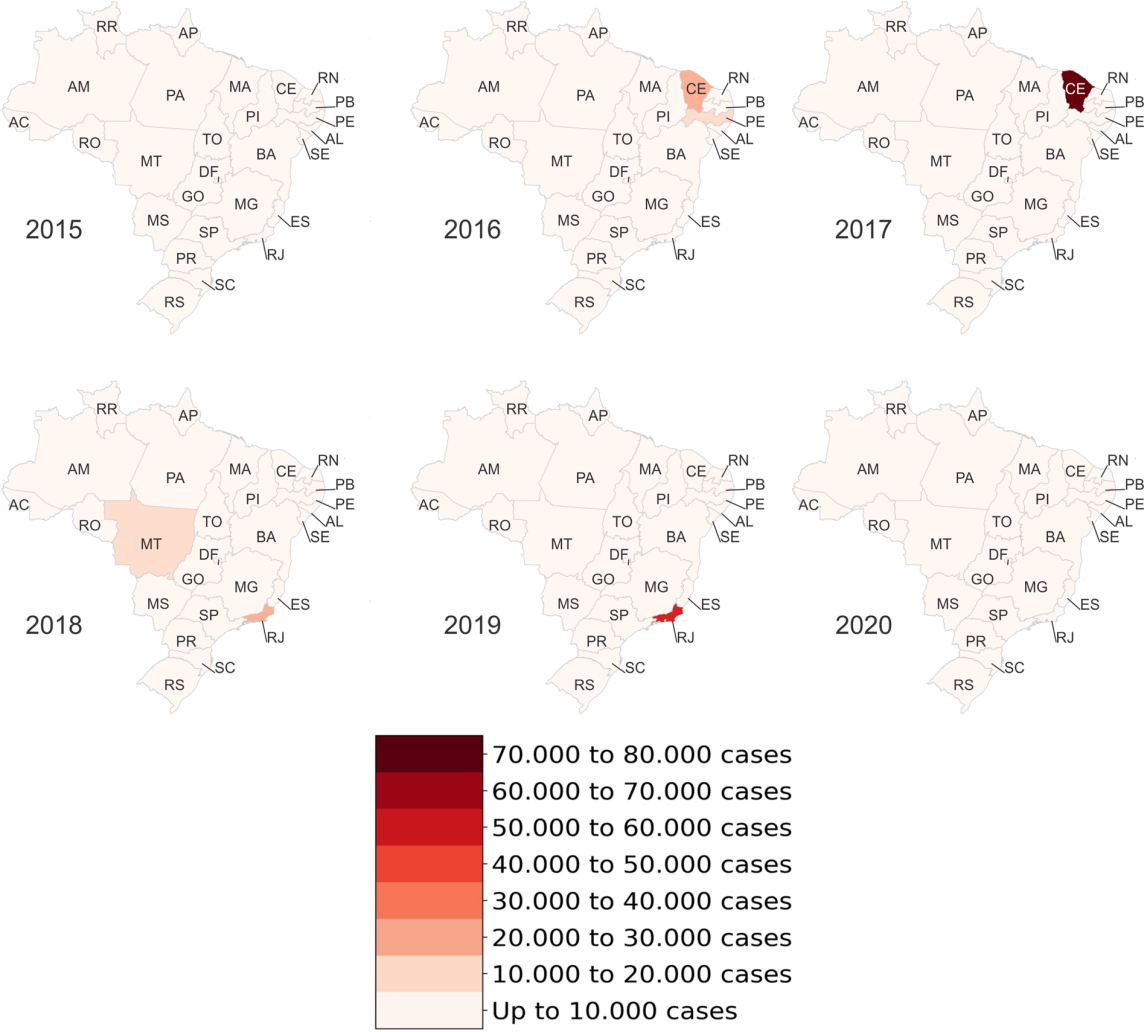
Occurrence of confirmed cases of Chikungunya by Brazilian state.

**Fig. 6:**
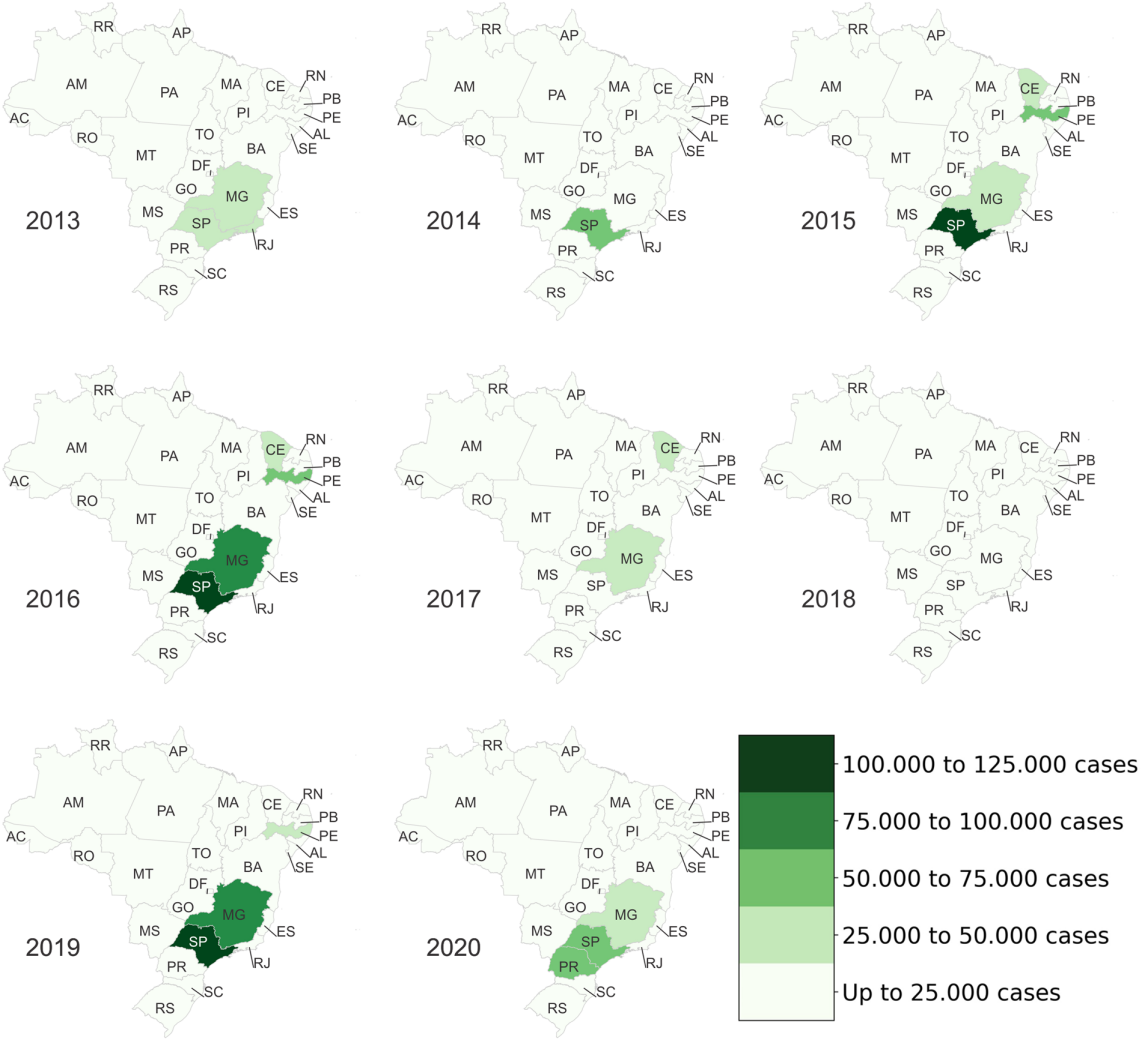
Occurrence of discarded/inconclusive cases of Dengue and Chikungunya by Brazilian state.

Figures 4–6 present heat maps of the number of Dengue, Chikungunya and discarded/inconclusive cases, respectively, by state and year. In these figures, the more intense the color, the greater the number of cases of each disease. Most Dengue cases (Fig. 4) occurred in the Southeast and Midwest of the country, more specifically in the states of Minas Gerais MG, Goiás GO and São Paulo SP. In 2015, SP had the highest number of cases of Dengue in a single state with more than 360,000 reported cases. This could reflect its population numbers and density.

Chikungunya emerged in the Americas in 2013^13^. Following the reporting of the first locally transmitted Chikungunya infection in Brazil in September 2014, the disease rapidly spread across Brazil^13^. Consistent with this timeline, the data set includes data for the years 2015 to 2020. Figure 5 illustrates the spread of Chikungunya in Brazil from the confirmation of initial autochthonous cases in Ceara CE in the Northeast to a major outbreak in Rio de Janeiro in 2018 and 2019.

Figure 6 shows discarded/inconclusive cases of Dengue and Chikungunya. Firstly, the number of cases is high in the states of CE and Pernambuco PE from 2015 to 2017, most likely reflecting the emergence of Chikungunya and associated difficulties in diagnosing the disease accurately^14^. This data raises questions regarding the quality of the surveillance system in these areas. For example, greater numbers of discarded/inconclusive cases in certain areas may indicate that the health and surveillance infrastructure in these areas is inferior to those in other states. Secondly, similar to Dengue, most of these categories of cases are located in the cities of SP and MG. Indeed, SP is the state with the highest number of cases in 2015, 2016 and 2019.

**Fig. 7:**
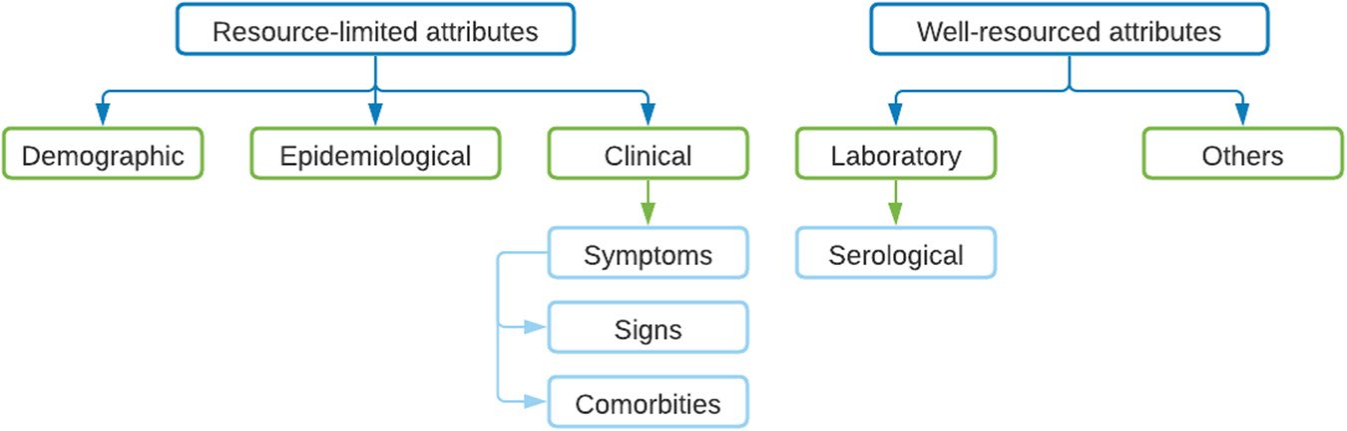
Attributes in the final data set.

The final data set is composed of 56 attributes that are grouped according to Fig. 7 and are detailed in Tables 2, 3, 4 and 5. Demographic, epidemiological and clinical (symptoms, signs and comorbidities) data were grouped as resource-limited attributes as per Lee *et al*.^15^. Specific equipment is not specified in the data set. Laboratory attributes (serological) and others are grouped as well-resourced attributes because they require specific equipment to be performed.

**Table 2:** Socio-demographic data.

Attribute	Description
ID_AGRAVO	ICD disease code
DT_NOTIFIC	Notification date
SEM_NOT	Epidemiological notification week
NU_ANO	Notification year
SG_UF_NOT	Acronym of the State of the health unit
ID_MUNICIP	City of Health Unit (IBGE Code)
ID_REGIONA	Health care regional code (where the health unit or other reporting source is located)
ID_UNIDADE	Health facility code
DT_SIN_PRI	Date of onset of severe symptoms
SEM_PRI	Epidemiological week of onset of symptoms
DT_NASC	Patient date of birth
NU_IDADE_N	Patient age
CS_SEXO	Patient sex
CS_GESTANT	Gestational Age of the Patient (Quarter) in case CS_SEXO = F
CS_RACA	Patient Race
CS_ESCOL_N	Patient education
SG_UF	Patient status (IBGE code)
ID_MN_RESI	City of the patient (IBGE code)
ID_RG_RESI	Health facility code
CS_ZONA	Area of Residence
ID_PAIS	Patient Country Code (IBGE Code)
DT_INVEST	Start date of case investigation
TPAUTOCTO	Indicates whether the case is indigenous to the area of residence.
COUFINF	State where the patient was infected (IBGE Code)
COPAISINF	Country where the patient was infected (IBGE Code)
COMUNINF	City where the patient was infected (IBGE Code)
EVOLUCAO	Case evolution
DT_ENCERRA	Case Closing Date

**Table 3:** Clinical data – Symptoms.

Attribute	Description
FEBRE	Symptom - Fever
MIALGIA	Symptom - Myalgia
CEFALEIA	Symptom - Headache
EXANTEMA	Symptom - Rash
VOMITO	Symptom - Vomiting
NAUSEA	Symptom - Nausea
DOR_COSTAS	Symptom - Back Pain
CONJUNTVIT	Symptom - Conjunctivitis
ARTRITE	Symptom - Arthritis
ARTRALGIA	Symptom - Arthralgia
PETEQUIA_N	Symptom - Petechiae
LACO	Symptom - Tourniquet test
DOR_RETRO	Symptom - Retro-orbital pain

**Table 4:** Clinical data – Comorbidities.

Attribute	Description
DIABETES	Pre-existing disease - Diabetes
HEMATOLOG	Pre-existing disease - Hematological disease
HEPATOPAT	Pre-existing disease - Liver disease
RENAL	Pre-existing disease - Kidney disease
HIPERTENSA	Pre-existing disease - Hypertension
ACIDO_PEPT	Pre-existing disease - Peptic acid disease
AUTO_IMUNE	Pre-existing disease - Autoimmune disease

**Table 5:** Laboratory data.

Attribute	Description
RESUL_SORO	Serological Test Results (IgM) Dengue
RESUL_NS1	Test Result Serology ELISA
RESUL_VI_N	Test Result Viral Isolation
RESUL_PCR_	RT-PCR Exam Result
HISTOPA_N	Histopathology Test Result
IMUNOH_N	Immunohistochemistry Test Result
HOSPITALIZ	If the patient was hospitalized
LEUCOPENIA	Leukopenia - Low level of white blood cells in the blood
CLASSI_FIN	Final patient classification

Socio-demographic data (Table 2) includes age, sex, gestational age, race, and area of residence, amongst others.

Symptoms relate to specific physical features which can indicate the existence of a disease. As per Table 3, the data set contains 13 symptoms.

Comorbidities are preexisting conditions in the patient. Table 4 presents the clinical data with information about comorbidities.

Table 5 presents the attributes for laboratory data. This data comprises results from serological and other tests. It also contains data on whether the patient was hospitalised as well as the final patient classification.

The general and disease baseline characteristics are shown in Table 6. Baseline characteristics show an overall mean (SD) age over 30 years and a predominance of women for each arboviral disease. Fever (37.3%), headache (34.5%), and myalgia (34%) were the most frequent symptoms. It is important to highlight that in confirmed cases of Chikungunya, the absence of symptoms in the records directly affect the percentage of these symptoms in general.

**Table 6:** General and disease baseline characteristics.

Variables	Total N = 6732542	Dengue N = 4307513	Chikungunya N = 325000	Others N = 2100029
Gender Women, %	3731577/6732542 (55.4)	2403184/4307513 (55.8)	194780/325000 (59.9)	1133495/2100029 (54)
Age, Mean (SD)	32 (18)	33 (18)	37 (20)	31 (18)
Race, (%)				
White	1,840,878 (27.3)	1,200,564 (27.9)	39,443 (12.1)	600,871 (28.6)
Black	243,673 (3.6)	155,374 (3.6)	14,505 (4.5)	73,794 (3.5)
Yellow	48,140 (0.7)	30,124 (0.7)	3,998 (1.2)	14,018 (0.7)
Admixed	2,277,168 (33.8)	1,341,361 (31.1)	170,074 (52.3)	765,733 (36.5)
Indigenous	15,484 (0.2)	10,246 (0.2)	691 (0.2)	4,547 (0.2)
Missing/ignored	2,307,199 (34.2)	1,569,844 (36.4)	96,289 (29,6)	641,066 (30.5)
Pregnant, (%)				
1st Quarter	13,641 (0.2)	7,915 (0.2)	910 (0.3)	4,816 (0.2)
2nd Quarter	17,463 (0.3)	10,007 (0.2)	1,505 (0.5)	5,951 (0.3)
3rd Quarter	14,223 (0.2)	7,951 (0.2)	1,204 (0.4)	5,068 (0.2)
Missing/ignored	6,687,215 (99.3)	4,281,640 (99.3)	321,381 (99)	2,084,194 (99.3)
Educational Degree, (%)				
Elementary School	587,216 (5.3)	229,742 (5.3)	15,434 (4.8)	116,632 (5.5)
Middle School	631,664 (9.3)	406,366 (9.4)	24,394 (7.5)	200,904 (9.6)
High School	1,093,285 (16.2)	698,230 (16.2)	37,686 (11.6)	357,369 (17.1)
College	265,913 (3.9)	168,808 (3.9)	8,495 (2.7)	88,610 (4.2)
Missing/ignored	4,342,024 (64.5)	2,781,507 (64.6)	236,702 (72.8)	1,323,815 (63)
Fever, (%)	2,508,024 (37.3)	1,714,334 (39.8)	139 (<0.1)	793,551 (37.8)
Myalgia, (%)	2,289,404 (34)	1,595,876 (37)	117 (<0.1)	693,411 (33)
Headache, (%)	2,325,434 (34.5)	1,611,029 (37.4)	115 (<0.1)	714,290 (34)
Rash, (%)	621,048 (9.2)	466,788 (10.8)	49 (<0.1)	154,211 (7.3)
Vomit, (%)	632,864 (9.4)	1,595,876 (37)	117 (<0.1)	693,411 (33)
Headache, (%)	2,325,434 (34.5)	1,611,029 (37.4)	115 (<0.1)	714,290 (34)
Rash, (%)	621,048 (9.2)	466,788 (10.8)	49 (<0.1)	154,211 (7.3)
Vomit, (%)	632,864 (9.4)	438,160 (10.2)	42 (<0.1)	194,662 (9.3)
Nausea, (%)	958,826 (14.2)	691,305 (16)	58 (<0.1)	267,463 (12.7)
Back pain, (%)	754,865 (11.2)	545,952 (12.7)	54 (<0.1)	208,859 (9.9)
Conjunctivitis, (%)	90,528 (1.3)	64,807 (1.5)	13 (<0.1)	25,708 (1.2)
Arthritis, (%)	288,109 (4.3)	214,337 (5)	30 (<0.1)	73,742 (3.5)
Arthralgia, (%)	635,375 (9.4)	451,362 (10.5)	58 (<0.1)	183,955 (8.8)
Petechiae, (%)	246,220 (3.7)	187,214 (4.3)	26 (<0.1)	58,980 (2.8)
Tourniquet test, (%)	119,836 (1.8)	97,642 (2.3)	5 (<0,1)	22,189 (1.1)
Retro-orbital pain, (%)	962,044 (14.3)	730,885 (17)	46 (<0.1)	231,113 (11)
Diabetes, (%)	63,657 (0.9)	45,088 (1)	8 (<0.1)	18,561 (0.9)
Hematological disease, (%)	12,701 (0.2)	8,751 (0.2)	1 (<0.1)	3,949 (0.2)
Liver disease, (%)	13,595 (0.2)	9,351 (0.2)	1 (<0.1)	4,243 (0.2)
Kidney disease, (%)	11,311 (0.2)	7,920 (0.2)	1 (<0.1)	3,390 (0.2)
Hypertension, (%)	156,779 (2.3)	112,685 (2.6)	12 (<0.1)	44,082 (2.1)
Peptic acid disease, (%)	14,842 (0.2)	10,258 (0.2)	2 (<0.1)	4,582 (0.2)
Autoimmune disease, (%)	11,318 (0.2)	8,031 (0.2)	0 (0)	3,287 (0.2)
Test Results (IgM) Dengue, (%)				
Positive	28,842 (0.4)	26,551 (0.6)	4 (<0.1)	2,287 (0,.)
Negative	49,175 (0.7)	19,659 (0.5)	13 (<0.1)	29,503 (1.4)
Inconclusive	13,381 (0.2)	7,387 (0.2)	2 (<0.1)	5,992 (0.3)
Not performed	6,641,144 (98.6)	4,253,916 (98.8)	324,981 (>99.9)	2,062,247 (98.2)
Test Result ELISA, (%)				
Positive	21,625 (0.3)	19,684 (0.5)	1 (<0.1)	1,940 (0.1)
Negative	137,247 (2)	51,030 (1.2)	1 (<0.1)	86,216 (4.1)
Inconclusive	2,659 (<0.1)	1,637 (<0.1)	0 (0)	1,022 (<0.1)
Not performed	6,571,011 (97.6)	4,235,162 (98.3)	324,998 (>99.9)	2,010,851 (95.8)
Test Result Viral Isolation, (%)				
Positive	207 (<0.1)	191 (<0.1)	0 (0)	16 (<0.1)
Negative	2,963 (<0.1)	2,036 (<0.1)	4 (<0.1)	923 (<0.1)
Inconclusive	909 (<0.1)	580 (<0.1)	0 (0)	329 (<0.1)
Not performed	6,728,463 (99.9)	4,304,706 (99.9)	324,996 (>99.9)	2,098,761 (99.9)
RT-PCR Exam Result, (%)				
Positive	670 (<0.1)	634 (<0.1)	0 (0)	36 (<0.1)
Negative	4,700 (0.1)	2,802 (0.1)	6 (<0.1)	1,892 (0.1)
Inconclusive	1,176 (<0.1)	731 (<0.1)	0 (0)	445 (<0.1)
Not performed	6,725,996 (99.9)	4,303,346 (99.9)	324,994 (>99.9)	2,097,656 (99.9)
Histopathology Test Result, (%)				
Positive	445 (<0.1)	404 (<0.1)	0 (0)	41 (<0.1)
Negative	1,677 (<0.1)	1,002 (<0.1)	1 (<0.1)	674 (<0.1)
Inconclusive	914 (<0.1)	566 (<0.1)	0 (0)	348 (<0.1)
Not performed	6,729,506 (>99.9)	4,305,541 (>99.9)	324,999 (>99.9)	2,098,966 (99.9)
Immunohistochemistry Test Result, (%)				
Positive	341 (<0.1)	309 (<0.1)	0 (0)	32 (<0.1)
Negative	2,165 (<0.1)	1,360 (<0.1)	1 (<0.1)	804 (<0.1)
Inconclusive	2,336 (<0.1)	1,519 (<0.1)	0 (0)	817 (<0.1)
Not performed	6,727,700 (99.9)	4,304,325 (99.9)	324,999 (>99.9)	2,098,376 (99.9)
Patient hospitalized, (%)	132,904 (2)	96,790 (2.2)	10 (<0.1)	36,104 (1.7)
Leukopenia, (%)	135,959 (2)	109,099 (2.5)	1 (<0.1)	26,859 (1.3)

Notes: (a) All data presented refers to suspected cases; (b) The classifications presented here here are in line with the Brazilian Ministry of Health guidelines; and (c) RT-PCR Exam Result refers to each specific virus defined in the respective column.

## Technical Validation

All data presented in this work can be corroborated by reports published by the Ministry of Health of Brazil.

## Usage Note

Robert *et al*.^16^ discuss the emergence of Dengue and related arboviruses (Zika and Chikungunya) in Córdoba, Argentina, and present a data set with records relating to the the transmission of Dengue, Chikungunya and Zika. This data set comprises data from 2009 to 2018 including known data on circulating dengue virus (DENV) serotypes and the origins of imported cases. In López *et al*.^17^, the Dengue outbreak in Santa Fé, Argentina was investigated. This city has a temperate climate and experienced an increase in Dengue cases and virus circulation from 2009. Santa Fé experienced the largest outbreak in Argentina to date. The intention of the authors of both papers was to support further research in understanding the factors and patterns of arboviruses emergence and transmission.

In line with Robert *et al*.^16^ and López *et al*.^17^, the data set presented in this work expands the data available to researchers on the emergence and transmission of two arboviruses, Dengue and Chikungunya. To this end, it complements these works and progresses work towards a potential international arbovirus data set suggested by Robert *et al*.^16^.

Arboviruses are hyperendemic in Brazil. The social, environmental and climate conditions in Brazil combined with disordered urban growth and population migration have escalated the public health risk presented by arboviruses. The COVID-19 pandemic and prolonged economic crisis are exacerbating efforts to control negative outcomes from these diseases^3^. These factors make it difficult to combat and prevent these diseases in the country, as well as to understand how the virus reacts and spreads. Although there is not complete data on all arboviruses, the data presented here can help in the fight against Dengue and Chikungunya, and assist in addressing misdiagnosis as experienced during the Zika epidemic in 2015^14^. For example, it can provide data develop (low cost) decision support tools for the differential diagnosis of these diseases. In particular, this data may be used as both training and test data sets for machine learning and deep learning models for binary and multi-class classification and prediction.

## Data Availability

The code used to pre-process the data set presented in this paper is available at: https://github.com/dotlab-brazil/arbovirus-dataset-brazil.
