# Author Correction: Arboviral disease record data - Dengue and Chikungunya, Brazil, 2013–2020

**DOI:** 10.1038/s41597-022-01397-0

**Published:** 2022-05-26

**Authors:** Sebastião Rogério da Silva Neto, Thomás Tabosa de Oliveira, Igor Vitor Teixiera, Leonides Medeiros Neto, Vanderson Souza Sampaio, Theo Lynn, Patricia Takako Endo

**Affiliations:** 1 Universidade de Pernambuco, Programa de Pós-Graduação em Engenharia da Computação, Recife, 50720-001 Brazil; 2 Fundação de Medicina Tropical Dr. Heitor Vieira Dourado, Manaus, 69040-000 Brazil; 3 Instituto Todos pela Saúde, São Paulo, 01310-942 Brazil; 4 Irish Institute of Digital Business, Dublin City University, Dublin, 9 Ireland

**Keywords:** Public health, Diseases

Correction to: *Scientific Data* 10.1038/s41597-022-01312-7, published online 10 May 2022

In this article the funding information relating to support from Coordenação de Aperfeiçoamento de Pessoal de Nível Superior - Brasil (CAPES) - Finance Code 001 was omitted. The original article has been corrected.

